# Genogroup I picobirnavirus in diarrhoeic foals: Can the horse serve as a natural reservoir for human infection?

**DOI:** 10.1186/1297-9716-42-52

**Published:** 2011-03-17

**Authors:** Balasubramanian Ganesh, Krisztian Banyai, Gisela Masachessi, Zornitsa Mladenova, Shigeo Nagashima, Souvik Ghosh, Seegekote Mariyappa Nataraju, Madhusudhan Pativada, Rahul Kumar, Nobumichi Kobayashi

**Affiliations:** 1 Division of Virology, National Institute of Cholera and Enteric Diseases (NICED), P-33, C.I.T. Road, Scheme-XM, Beliaghata, Kolkata 700 010, West Bengal, India; 2 Veterinary Medical Research Institute, Hungarian Academy of Sciences, Hungaria krt. 21, 1143 Budapest, Hungary; 3 Institute of Virology "Dr. J. M. Vanella", Faculty of Medical Sciences, National University of Cordoba, Cordoba, Argentina; 4 Department of Virology, National Reference Laboratory of Enteroviruses, National Center of Infectious and Parasitic Diseases, 44A, Stoletov Blvd., Sofia 1233, Bulgaria; 5 Division of Virology, Department of Infection and Immunity, Jichi Medical University School of Medicine, 3311-1 Yakushiji, Shimotsuke-shi, Tochigi-ken, 329-0498, Japan; 6 Department of Hygiene, Sapporo Medical University School of Medicine, S-1 W-17, Chuo-Ku, Sapporo 060-8556, Japan

## Abstract

Picobirnaviruses (PBV) are small, non-enveloped viruses with a bisegmented double-stranded RNA genome. In this study a PBV strain, PBV/Horse/India/BG-Eq-3/2010, was identified in the faeces of a 10 month old weaned female foal with diarrhoea in January 2010 from Kolkata, India. Surprisingly, sequence comparison and phylogenetic analysis of a short stretch of the RNA dependent RNA polymerase gene revealed close genetic relatedness (> 98% nucleotide identity) to a human genogroup I PBV strain (Hu/GPBV1) detected earlier from the same part of India. Our observations together with earlier findings on genetic relatedness between human and animal PBV warrant further studies on zoonotic potential.

## Introduction, Methods and Results

Picobirnaviruses (PBV) are small, non-enveloped viruses, with a bisegmented dsRNA genome. PBV exists as either a large genome profile (2.3 to 2.6 kbp and 1.5 to 1.9 kbp for the segments 1 and 2, respectively) [1-3] or small genome profile (1.75 kbp and 1.55 kbp for the segments 1 and 2, respectively) [4,5]. The PBV capsid protein has two main domains and forms a simple, spherical 33-41 nm virion [6].

The genomic segment 1 of the prototype PBV strain, Hy005102 was found to encode two open reading frames (ORF1 and ORF2) [7]; ORF1 codes for a hydrophilic group of 224 amino acids of unknown function. ORF2 has 552 amino acids that encode the capsid protein. The smaller segment 2 has a single ORF of 534 amino acids encoding the viral RNA dependent RNA polymerase (RdRp). Recently, additional sequence information of full or nearly full genome segments has been made available in the public DNA databases for other human strains [8,9] as well as a lapin [10] and a bovine PBV strain [11].

Historically, PBV were first detected in the faecal specimens of humans and free-living rats (*Oryzomys nigripes*) in 1988 from Brazil [1,12]. Thereafter, PBV were detected in human faeces [7-9,13-16] and in a wide range of animal species worldwide. Farm mammals such as pigs, calves, foals, lambs, avians such as chickens, free-living mammals, companion and zoo animals, a variety of wild birds and even snakes have been found to shed PBV with or without diarrhoea [10,11,17-26]. The lack of a consistent pattern in PBV detection concomitantly in healthy [27] and diarrhoeic individuals and animal species raises questions regarding the pathogenic potential of PBV, albeit they have been often implicated in opportunistic infections in immunocompromised patients [28-30].

The detection of the bisegmented dsRNA genome of PBV by polyacrylamide gel electrophoresis (PAGE) and silver staining [31], is one of the standard and reliable laboratory diagnoses. Broadly reactive primer pairs for RT-PCR [8] have served as an alternative to PAGE, for molecular detection and characterisation of PBV. These RT-PCR primers specifically amplify small fragments within the RdRp gene, and are also capable of differentiating 2 major PBV genogroups, genogroup I and genogroup II. These primer sets were utilised in the present study, where we report the detection and molecular characterisation of an equine picobirnavirus detected in a diarrhoeic foal. Further characterisation by sequencing and phylogenetic analyses revealed that this equine PBV strain belongs to genogroup I and clusters with an Indian human and some Hungarian porcine PBV strains.

Seven faecal specimens were collected from adult domestic horses and foals (*Equus ferus caballus*) with or without diarrhoea from Kolkata, India during January 2010 as part of an ongoing study on picobirnavirus infections (Table 1): two from foals, two from colts, and one each from a filly, a mare and a stallion. The age of hosts ranged from 9 to 72 months.

**Table 1:** Details of faecal specimens collected from domestic horses and foals in Kolkata, India during January 2010 and screening for rotavirus and picobirnavirus by PAGE and RT-PCR assay.

**Sl. No**.	Sample code	Age and Sex	Health status	Stool consistency	PAGE Screening		RT-PCR	
					
					RV	PBV	RV	PBV
1.	BG-Eq-1	3 yr/F (Filly)	Healthy	Normal	-	-	-	-
2.	BG-Eq-2	5 yr/F (Mare)	Diarrhoeic	Loose	-	-	-	-
3.	BG-Eq-3	10 m/F (Foal)	Diarrhoeic	Loose	-	-	-	(+) ve 201 bp GGI
4.	BG-Eq-4	3 yr/M (Colt)	Healthy	Normal	-	-	-	-
5.	BG-Eq-5	6 yr/M (Stallion)	Healthy	Normal	-	-	-	-
6.	BG-Eq-6	9 m/M (Foal)	Healthy	Normal	-	-	-	-
7.	BG-Eq-7	3 yr/M (Colt)	Healthy	Normal	-	-	-	-

From 10% fecal suspensions, total RNA was extracted for PAGE and silver staining [14,31]. Subsequently, the viral RNA was amplified using the primers and algorithms described previously [8,14]. The amplicons were purified and sequenced in both directions. The algorithm of nucleotide sequence editing and phylogenetic analysis was done as previously described [14,15]. The nucleotide sequence of the foal PBV strain, PBV/Horse/India/BG-Eq-3/2010, was deposited in the DNA database (accession no. DDBJ: AB598401).

Screening of faecal RNA extracts by PAGE gave negative results. (Similarly, RT-PCR assay using the primers [32] targeting the rotavirus VP4 and VP7 genes failed to detect the group A rotavirus). However, a single faecal specimen obtained from a 10 month old weaned female foal was found to be positive for genogroup I PBV by RT-PCR using the genogroup specific primer pairs [8]. Sequence analysis of the amplicon indicated the presence of genogroup I PBV strain. This PBV strain, PBV/Horse/India/BG-Eq-3/2010, shared 70% nt and 66% aa identity with the prototype genogroup I PBV strain, 1-CHN-97 and only about 24% aa similarity to the prototype genogroup II PBV strain, 4-GA-91.

The nucleotide identities along a 170 bp stretch and the partial length deduced amino acid identity (given in parentheses) of gene segment 2 (stretch of 56 amino acids) between the equine genogroup I PBV strain (PBV/Horse/India/BG-Eq-3/2010) detected in Kolkata, India and the related human and porcine PBV strains were compared (Additional file 1: Table S1). In general, similarity values fell in the same range as described for other genogroup I PBV strains in some recent studies [15] except for a single Indian (Kolkata) human PBV strain GPBV1 [15] (98% nt identity) and a few Hungarian porcine PBV strains [3] Po/D4/C-5/Hun; Po/D4/C-1/Hun; Po/D6/C-19/Hun; and Po/C6/C-17/Hun (75-87% nt identity). In addition, the deduced stretch of 56 amino acids of our equine PBV strain and the majority of genogroup I PBV strains showed that 16 amino acids were conserved (Additional file 2: Table S2).

The "proline" in aa position 13 (the position in the alignment used for comparison) is conserved in all the PBV strains analysed to date, whereas another proline residue in aa position 25 was conserved in all other species except in bovine PBV strain Bo/RUBV-P. Similarly, the "methionine" in aa position 30 was conserved in all but two PBV strains: the human strain VS-22 identified from the Netherlands and the bovine strain RUBV-P from India.

**Figure 1:**
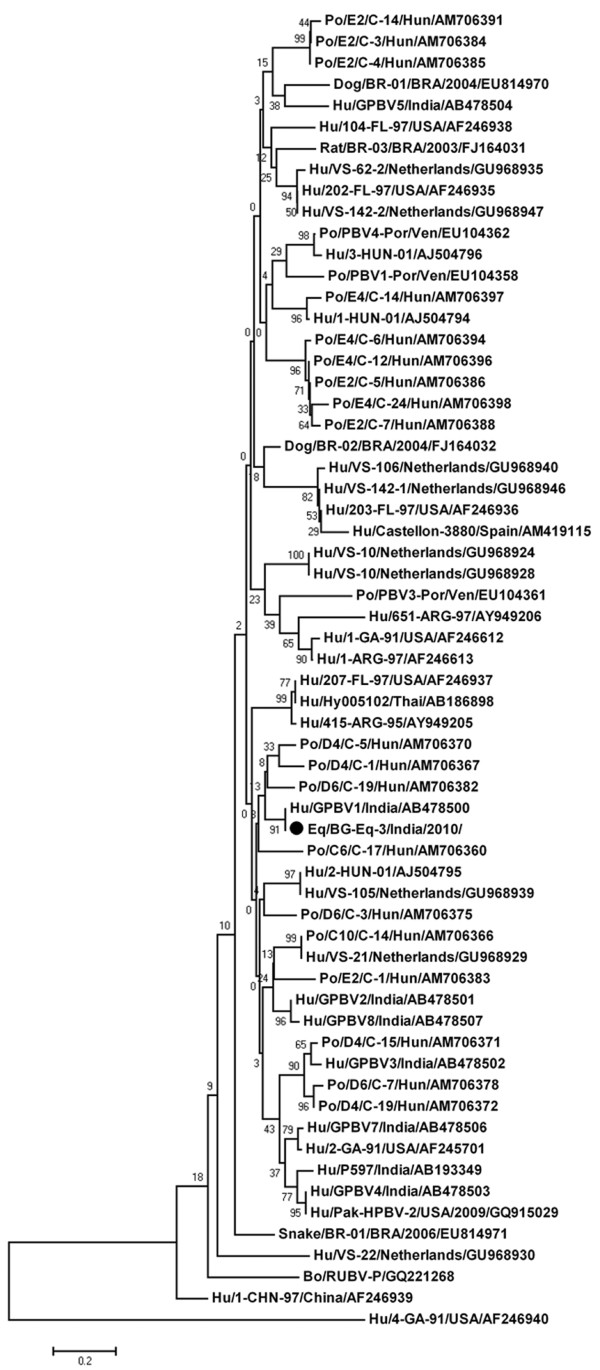
**Phylogenetic tree showing the equine picobirnavirus strain (Genogroup I PBV/Horse/India/BG-Eq-3/2010) with cognate stretch of hitherto reported human, porcine, bovine, canine, murine and serpentine genogroup I PBV strains, based on partial amino acid sequence (56 amino acids (aa)) of genomic segment 2**. The phylogenetic tree was constructed by the neighbor-joining method using the MEGA software (Version 4.1). Phylogenetic distances were measured by the Kimura two-parameter model, and the tree was statistically supported by bootstrapping with 1000 replicates. The genogroup I equine PBV strain BG-Eq-3 is denoted with a ● symbol. The tree was rooted with cognate stretch of gene segment 2 of genogroup II prototype strain Hu/4-GA-91 (USA) defined as the outgroup strain. Abbreviations: Hu, Human; Po, Porcine; Bo, Bovine; Eq, Equine; ARG, Argentina; BRA, Brazil; Hun, Hungary; Thai, Thailand; USA, United States of America; Ven, Venezuela.

Subsequently, the phylogenetic tree was constructed with a cognate stretch of hitherto reported human, porcine, bovine, canine, murine and serpentine genogroup I PBV strains (Figure 1) based on partial amino acid sequence (56 amino acids) of genomic segment 2. This analysis revealed that the PBV/Horse/India/BG-Eq-3/2010 strain showed very close evolutionary relatedness to the Indian (Kolkata) human PBV strain GPBV1 [15] (100% aa identity) and a few Hungarian porcine PBV strains [17] Po/D4/C-5/Hun; Po/D4/C-1/Hun; Po/D6/C-19/Hun; and Po/C6/C-17/Hun (77-84% aa identity).

## Discussion

PBV in horses has been reported earlier [18], however this is the first description of an equine PBV strain at the molecular level. Since, the number of samples taken for this study was very small, this proportion cannot be taken as an indication of prevalence or incidence of PBV infection or disease association in domestic horses in Kolkata, India. The length of the gene fragment used for characterisation of PBV strains was relatively short, thus conclusions on the evolutionary relationships between any two PBV may have limitations.

In addition, PBV have 2 genome segments and there is a theoretic possibility that genome segment reassortment may permit a large variety of the RdRp and capsid gene combinations to rise. Despite these shortcomings, this is the first study that provides evidence for genetic relationship between human and equine PBV strains.

One alternative to explain the close genetic relationship between these heterologous strains is the common exposure to PBV infection. PBV are commonly detected in communal sewage and surface waters [33], thus consumption of water contaminated with PBV may provide one way to acquire PBV by different host species. Another possible explanation is that one host may serve as source of infection for PBV to be transmitted to the other host. Whether horses or humans are the primary hosts for the particular strain that was found in this study and in another study [15] remains to be carefully investigated. Nonetheless, genogroup I PBV detected in pigs in parts of Europe [17] and Latin America [20] were closely related to human genogroup I PBV, suggesting the zoonotic potential of PBV strains.

Partial molecular characterisation and sequence analysis of human and animal PBV strains have shown that distinct sequence heterogeneity exists among PBV, implicating the importance of continued surveillance for newly emerging variants. Full genome analyses of the strains identified in the future may reveal the evolutionary mechanisms of PBV, pointing out the role of genetic drift and reassortment on the overall genomic architecture and to the possible traits implicated in the host range.

## List of Abbreviations

PBV: picobirnavirus; dsRNA: double-stranded RNA; kbp: kilo base pair; ORF: open reading frame; RdRp: RNA dependent RNA polymerase; PAGE: polyacrylamide gel electrophoresis; RT-PCR: reverse transcription-polymerase chain reaction; nt: nucleotide; aa: amino acid; Hu: human; Po: porcine; Bo: bovine; Eq: equine

## Competing interests

The authors declare that they have no competing interests.

## Authors' contributions

GB: conceived of the study, study design and coordination, performed laboratory assays, drafted and revised the manuscript. KB: analysed and interpreted the data, drafted and revised the manuscript. MG; MZ: participated in preparation of illustrations, revising the draft for important intellectual content. NS; GS; KN: participated in the design of the study, drafting the manuscript. NSM; PM; KR: participated in the molecular assays and sequence analysis. All the authors read and approved the final manuscript.

## Supplementary Material

Additional file 1 **Table S1**: Comparison of the percentage nucleotide identity and (amino acid identity given in parentheses) of Equine Picobirnavirus detected in a diarrhoeic foal in Kolkata, India with some of the hitherto reported human, porcine, canine, murine, bovine and serpentine picobirnaviruses. Click here for file

Additional file 2 **Table S2**: Comparison of partial length deduced amino acid sequence of segment 2 of the Equine picobirnavirus detected in Kolkata with the hitherto reported human, porcine, canine, murine, bovine and serpentine picobirnaviruses. Click here for file
