# Supplementary material for: Genogroup I picobirnavirus in diarrhoeic foals: Can the horse serve as a natural reservoir for human infection?
Source: Vet Res. 2011 Mar 17;42(1):52. doi: 10.1186/1297-9716-42-52 (PMC3068956; doi:10.1186/1297-9716-42-52)
Supplement: Additional file 1 — Table S1: Comparison of the percentage nucleotide identity and (amino acid identity given in parentheses) of Equine Picobirnavirus detected in a diarrhoeic foal in Kolkata, India with some of the hitherto reported human, porcine, canine, murine, bovine and serpentine picobirnaviruses. [file 1297-9716-42-52-S1.DOC]

|  | Eq/BG-Eq-3 | Hu/  GPBV1 | Po/D6  /C-19 | Po/C6  /C-17 | Hu/2-HUN-01 | Hu/Pak-HPBV2 | Hu/1-CHN-97 | Hu/Hy005102 | Hu/Castellon-3880 | Hu/  VS-22 | Po/PBV3-Por | Dog/  BR-02 | Rat/  BR-03 | Bo/RUBV-P | Snake/  BR-01 |
| --- | --- | --- | --- | --- | --- | --- | --- | --- | --- | --- | --- | --- | --- | --- | --- |
| Eq/BG-Eq-3/  India |  | 98.2[--] | 87.0  [83.9] | 74.9  [76.8] | 67.7  [75.0] | 73.0  [75.0] | 69.9  [66.1] | 70.1  [73.2] | 61.6  [63.8] | 64.9  [60.7] | 64.1  [60.7] | 70.2  [71.4] | 62.5  [63.0] | 61.0  [57.1] | 69.1  [72.0] |
| Hu/GPBV1/  AB478500 |  |  | 85.1  [83.9] | 73.7  [76.8] | 67.1  [75.0] | 72.8  [75.0] | 69.3  [66.1] | 69.6  [73.2] | 60.1  [63.8] | 63.5  [60.7] | 63.5  [60.7] | 69.1  [71.4] | 61.9  [63.0] | 61.0  [57.1] | 69.1  [72.0] |
| Po/D6/C-19/  AM706382 |  |  |  | 71.9  [75.0] | 68.5  [73.2] | 73.2  [76.8] | 66.3  [62.5] | 72.5  [75.0] | 62.3  [61.7] | 63.1  [64.3] | 65.0  [60.7] | 68.9  [76.8] | 65.7  [68.5] | 63.4  [58.9] | 67.1  [72.0] |
| Po/C6/C-17/  AM706360 |  |  |  |  | 67.3  [75.0] | 72.5  [73.2] | 57.5  [55.4] | 68.9  [76.8] | 66.9  [66.0] | 64.7  [53.6] | 62.9  [60.7] | 70.7  [75.0] | 60.5  [61.1] | 58.7  [50.0] | 74.3  [70.0] |
| Hu/2-HUN-01/  AJ504795 |  |  |  |  |  | 70.3  [69.6] | 63.3  [57.1] | 71.0  [73.2] | 78.6  [72.9] | 63.8  [51.8] | 61.5  [57.1] | 67.9  [73.2] | 65.9  [64.8] | 58.1  [57.1] | 70.5  [72.0] |
| Hu/Pak-HPBV-2/  GQ915029 |  |  |  |  |  |  | 63.0  [62.5] | 67.1  [71.4] | 68.3  [63.8] | 65.5  [62.5] | 62.5  [60.7] | 71.3  [75.0] | 61.0  [64.8] | 60.5  [58.9] | 68.8  [72.9] |
| Hu/1-CHN-97/  AF246939 |  |  |  |  |  |  |  | 65.7  [58.9] | 60.7  [57.4] | 61.6  [58.9] | 64.8  [55.4] | 64.6  [67.9] | 60.4  [53.7] | 56.0  [55.4] | 68.8  [62.0] |
| Hu/Hy005102/  AB186898 |  |  |  |  |  |  |  |  | 61.9  [63.8] | 64.3  [58.9] | 63.5  [64.3] | 70.2  [75.0] | 67.1  [63.0] | 59.5  [57.1] | 72.1  [70.0] |
| Hu/Castellon-3880/AM419115 |  |  |  |  |  |  |  |  |  | 66.2  [55.3] | 61.8  [59.2] | 76.1  [76.6] | 71.7  [60.0] | 55.1  [45.8] | 71.1  [61.0] |
| Hu/VS-22/  GU968930 |  |  |  |  |  |  |  |  |  |  | 66.3  [50.0] | 63.5  [60.7] | 65.6  [55.6] | 58.8  [51.8] | 65.6  [56.0] |
| Po/PBV3-Por/  EU104361 |  |  |  |  |  |  |  |  |  |  |  | 65.7  [66.1] | 64.4  [57.4] | 55.0  [48.2] | 67.2  [62.0] |
| Dog/BR-02/  FJ164032 |  |  |  |  |  |  |  |  |  |  |  |  | 67.5  [72.2] | 58.2  [55.4] | 72.5  [74.0] |
| Rat/BR-03/  FJ164031 |  |  |  |  |  |  |  |  |  |  |  |  |  | 61.3  [57.4] | 73.3  [69.2] |
| Bo/RUBV-P/  GQ221268 |  |  |  |  |  |  |  |  |  |  |  |  |  |  | 60.4  [56.0] |
| Snake/BR-01/  EU814971 |  |  |  |  |  |  |  |  |  |  |  |  |  |  |  |

Supplementary Table S1: Comparison of the percentage nucleotide identity and (amino acid identity given in parentheses) of Equine Picobirnavirus detected in a diarrhoeic foal in Kolkata, India with some of the hitherto reported human, porcine, canine, murine, bovine and serpentine picobirnaviruses.
