# Supplementary material for: Genogroup I picobirnavirus in diarrhoeic foals: Can the horse serve as a natural reservoir for human infection?
Source: Vet Res. 2011 Mar 17;42(1):52. doi: 10.1186/1297-9716-42-52 (PMC3068956; doi:10.1186/1297-9716-42-52)
Supplement: Additional file 2 — Table S2: Comparison of partial length deduced amino acid sequence of segment 2 of the Equine picobirnavirus detected in Kolkata with the hitherto reported human, porcine, canine, murine, bovine and serpentine picobirnaviruses. [file 1297-9716-42-52-S2.DOC]

Supplementary Table S2. Comparison of partial length deduced amino acid sequence of segment 2 of the Equine picobirnavirus detected in Kolkata with the hitherto reported human, porcine, canine, murine, bovine and serpentine picobirnaviruses.

| **AA position** | 1 | 2 | 3 | 5 | 6 | 8 | 9 | 10 | 14 | 15 | 16 | 17 | 18 | 19 | 20 | 21 | 22 | 23 | 24 | 25 | # | # | # | # | 31 | # | 35 | # | 37 | # | # | # | # | 45 | # | 47 | # | # | 50 | 51 | 56 |
| --- | --- | --- | --- | --- | --- | --- | --- | --- | --- | --- | --- | --- | --- | --- | --- | --- | --- | --- | --- | --- | --- | --- | --- | --- | --- | --- | --- | --- | --- | --- | --- | --- | --- | --- | --- | --- | --- | --- | --- | --- | --- |
| **Hu/1-CHN-97** | **F** | **G** | **V** | **V** | **K** | **L** | **Q** | **F** | **A** | **I** | **E** | **I** | **A** | **Q** | **K** | **R** | **W** | **I** | **T** | **P** | **A** | **I** | **G** | **M** | **E** | **A** | **Q** | **R** | **I** | **K** | **L** | **D** | **T** | **A** | **K** | **S** | **E** | **L** | **V** | **I** | **S** |
| **Hu/VS-22/NL** | **.** | **T** | **.** | **L** | **Q** | **.** | **R** | **V** | **F** | **M** | **D** | **M** | **L** | **.** | **.** | **H** | **K** | **.** | **I** | **.** | **.** | **V** | **G** | **L** | **.** | **E** | **N** | **K** | **.** | **.** | **.** | **.** | **.** | **G** | **E** | **D** | **D** | **V** | **.** | **.** | **.** |
| **Eq/BG-Eq-3/IND** | **.** | **S** | **.** | **I** | **N** | **.** | **.** | **V** | **L** | **.** | **.** | **S** | **C** | **.** | **.** | **F** | **D** | **L** | **V** | **.** | **.** | **V** | **S** | **.** | **.** | **S** | **.** | **.** | **.** | **M** | **M** | **.** | **.** | **.** | **.** | **D** | **D** | **V** | **.** | **.** | **.** |
| **Hu/GPBV1/IND** | **.** | **S** | **.** | **I** | **N** | **.** | **.** | **V** | **L** | **.** | **.** | **S** | **C** | **.** | **.** | **F** | **D** | **L** | **V** | **.** | **.** | **V** | **S** | **.** | **.** | **S** | **.** | **.** | **.** | **M** | **M** | **.** | **.** | **.** | **.** | **D** | **D** | **V** | **.** | **.** | **.** |
| **Po/D6/Cl-19/HUN** | **.** | **A** | **.** | **I** | **C** | **.** | **.** | **V** | **L** | **.** | **.** | **A** | **C** | **.** | **.** | **F** | **N** | **L** | **I** | **.** | **.** | **V** | **S** | **.** | **.** | **S** | **.** | **.** | **V** | **.** | **M** | **.** | **.** | **G** | **T** | **D** | **D** | **V** | **.** | **.** | **.** |
| **Hu/Pak-HPBV-2** | **.** | **A** | **.** | **I** | **Q** | **.** | **R** | **L** | **L** | **.** | **.** | **T** | **C** | **.** | **.** | **F** | **N** | **L** | **V** | **.** | **.** | **V** | **S** | **.** | **.** | **.** | **K** | **.** | **.** | **.** | **.** | **.** | **.** | **S** | **A** | **D** | **D** | **D** | **I** | **.** | **.** |
| **Po/C6/Cl-17/HUN** | **.** | **A** | **.** | **I** | **R** | **.** | **.** | **M** | **L** | **.** | **.** | **C** | **F** | **R** | **S** | **F** | **D** | **L** | **V** | **.** | **.** | **V** | **S** | **.** | **.** | **S** | **K** | **.** | **.** | **R** | **M** | **.** | **.** | **G** | **D** | **D** | **D** | **D** | **.** | **V** | **.** |
| **Hu/2-HUN-01/HUN** | **Y** | **A** | **.** | **I** | **E** | **.** | **S** | **V** | **L** | **.** | **.** | **K** | **V** | **.** | **S** | **F** | **N** | **L** | **V** | **.** | **.** | **V** | **S** | **.** | **.** | **S** | **R** | **.** | **.** | **A** | **M** | **.** | **.** | **.** | **S** | **N** | **D** | **.** | **.** | **V** | **.** |
| **Hu/Hy005102/Thai** | **.** | **A** | **.** | **I** | **R** | **.** | **.** | **V** | **L** | **.** | **L** | **T** | **F** | **.** | **R** | **L** | **G** | **L** | **V** | **.** | **.** | **V** | **S** | **.** | **.** | **.** | **R** | **.** | **.** | **.** | **M** | **.** | **.** | **G** | **P** | **R** | **D** | **V** | **.** | **V** | **.** |
| **Dog/BR-02/BRA** | **.** | **A** | **.** | **.** | **R** | **.** | **.** | **V** | **L** | **.** | **.** | **A** | **.** | **.** | **.** | **F** | **N** | **L** | **V** | **.** | **.** | **V** | **S** | **.** | **D** | **E** | **K** | **.** | **.** | **.** | **.** | **.** | **.** | **G** | **Q** | **.** | **D** | **.** | **.** | **V** | **.** |
| **Po/PBV3-Por/VEN** | **.** | **.** | **I** | **I** | **L** | **H** | **.** | **V** | **L** | **T** | **.** | **A** | **F** | **K** | **R** | **F** | **G** | **Y** | **V** | **.** | **.** | **V** | **S** | **.** | **D** | **.** | **L** | **E** | **.** | **.** | **.** | **.** | **S** | **D** | **D** | **K** | **D** | **.** | **.** | **.** | **.** |
| **Rat/BR-03/BRA** | **Y** | **A** | **.** | **I** | **A** | **.** | **.** | **V** | **L** | **.** | **.** | **A** | **.** | **.** | **N** | **T** | **R** | **L** | **I** | **.** | **S** | **V** | **S** | **.** | **D** | **E** | **R** | **A** | **.** | **Y** | **.** | **.** | **.** | **G** | **R** | **D** | **D** | **.** | **I** | **.** | **,,** |
| **Snake/BR-01/BRA** | **.** | **A** | **.** | **I** | **A** | **.** | **.** | **V** | **M** | **.** | **.** | **A** | **.** | **.** | **R** | **F** | **D** | **L** | **V** | **.** | **.** | **V** | **G** | **.** | **.** | **S** | **R** | **.** | **.** | **.** | **.** | **.** | **N** | **S** | **S** | **D** | **D** | **.** | **.** | **,,** | **,,** |
| **Bo/RUBV-P/IND** | **.** | **.** | **.** | **I** | **A** | **.** | **S** | **V** | **L** | **.** | **.** | **R** | **C** | **.** | **D** | **K** | **N** | **L** | **I** | **A** | **P** | **I** | **G** | **L** | **S** | **.** | **R** | **.** | **.** | **Q** | **.** | **E** | **.** | **R** | **P** | **E** | **D** | **Y** | **.** | **.** | **T** |

F G V **N** V K **E** L Q F **Y Q P*** A I E I A Q K R W I T **P*** A **W** I G M E A **V D** Q R I **T** K L **F** D T **K** A K S E L V I **C T D F** S

The conserved proline^*^ residue (aa13 and aa25) and other amino acids (aa4; aa7; aa11; aa12; aa13; aa25; aa27; aa33; aa34; aa38; aa41; aa44; aa52; aa53; aa54; and aa55) are underlined and shown in bold face.
